# Clinical Diagnosis of Toxoplasmosis via Histopathological and Antibody Examination of Five Immunocompetent Patients at Kobe City Medical Center General Hospital, 2008 to 2015

**DOI:** 10.1155/2021/6611273

**Published:** 2021-10-12

**Authors:** Tatsuji Hoshino, Shinya Yoshioka, Shogo Shinohara, Akiko Matsushita, Yukihiro Imai, Masato Kinugasa, Yoshiyuki Tsuji

**Affiliations:** ^1^Meiwa General Hospital, Department of Obstetrics and Gynecology, Nishinomiya, Japan; ^2^Kobe City Medical Center General Hospital, Department of Obstetrics and Gynecology, Kobe, Japan; ^3^Kobe City Medical Center General Hospital, Department of Head and Neck Surgery, Kobe, Japan; ^4^Kobe City Medical Center General Hospital, Department of Hematology, Kobe, Japan; ^5^Kobe City Medical Center General Hospital, Division of Clinical Pathology, Kobe, Japan

## Abstract

Congenital toxoplasmosis, commonly known as TORCH, is a well-known syndrome, but even experienced obstetricians rarely encounter it. In Japan, there is good overall hygiene and raw or wild game meats are not eaten; therefore, the prevalence of *Toxoplasma gondii* infection and the antibody positivity rates have been low. This low prevalence rate also relates to the fact that *Toxoplasma gondii* infections are rarely observed in immunocompetent hosts. Exploration of the cases in which pathological examinations were performed at our hospital (Kobe City Medical Center General Hospital) revealed that acquired Toxoplasma infections were apparent in five immunocompetent patients over an 8-year period. The number of infections was unexpectedly high. The number of 5 cases was the highest in literature review to the extent that we could know. To prevent congenital toxoplasmosis, which manifests as intracranial calcifications, hydrocephalus, and chorioretinitis in severe cases, pregnant women and their doctors require proper knowledge about the risk factors and danger of this infection. We believe that from the viewpoint of cost performance relationship, it is appropriate to bear the test fee of about 50 USD for Toxoplasma IgG and IgM check for the test of congenital toxoplasmosis, if patients desired.

## 1. Introduction

Toxoplasmosis is an infection caused by *Toxoplasma gondii*. Congenital toxoplasmosis, also known as TORCH syndrome [1], is rarely encountered even by experienced obstetricians [2]. We previously experienced and reported a case of congenital toxoplasmosis stillbirth diagnosed with autopsy [3]. However, there were no patients being treated for Toxoplasma chorioretinitis, Toxoplasma hydrocephalus, or Toxoplasma mental retardation in our hospital. It appears that *Toxoplasma gondii* infections in non-pregnant immunocompetent hosts occur more frequently than congenital toxoplasmosis. We examined the cases in our hospital and sought to determine how many Toxoplasma infections had occurred recently. By examining the cases in our hospital, we considered whether infection with *Toxoplasma gondii* has occurred rather more often than we initially anticipated.

## 2. Methods

A Toxoplasma antibody test was performed along with histopathological investigations at our hospital (Kobe City Medical Center General Hospital, Japan) when a *Toxoplasma gondii* infection was suspected in a patient. When a case was positive for both Toxoplasma-specific IgG and IgM antibodies, we clinically diagnosed the case as having a *Toxoplasma gondii* infection. There were five clinically diagnosed patients. One pregnant patient had an intrauterine fetal death, and a Toxoplasma cyst was observed in the autopsy specimen, placenta, fetal heart, adrenal gland, and brain. Four cases had superficial lymphadenopathy, and pathological examination was performed to differentiate malignant lymphoma and metastatic tumor from non-specific adenopathy. Hematoxylin and eosin staining examination of the samples revealed the three main features of Toxoplasma lymphadenitis, namely, reactive follicles, epithelioid cell clusters, and patches of monocytoid cells [4].

## 3. Results

**Figure 1:**
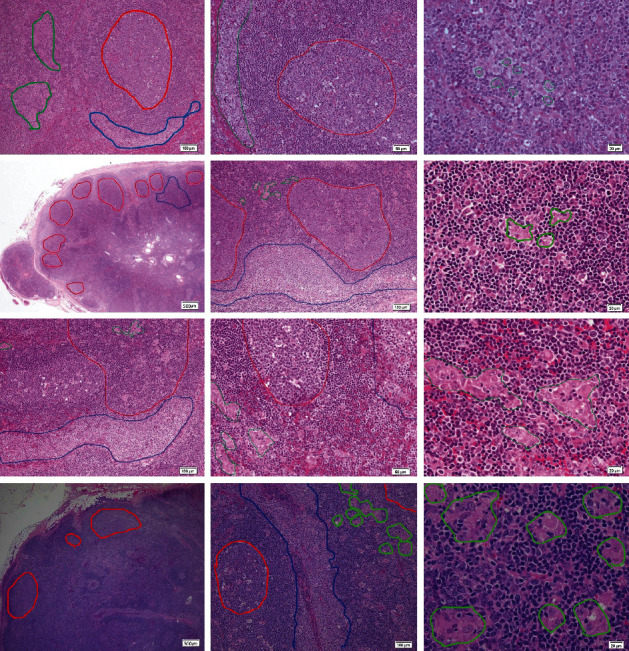
Microscopic findings of hematoxylin-eosin-stained lymph nodes in four cases of Toxoplasma infection (cases 2–5). Case 2: reactive follicles (red line circle), clusters of epithelioid cells (histiocytes, green line circle), and patches of monocytoid cells (monocyte-like cells, blue line circle); (hematoxylin-eosin stain) the first row, left: ×100 (microscope total magnification), mid: ×200, and right: ×400. Case 3: reactive follicles (red line circle), epithelioid cell clusters (histiocytes, green line circle), and monocytoid cell patches (monocyte-like cells, blue line circle); (hematoxylin-eosin stain) the second row, left: ×20, mid: ×100, and right: ×400. Case 4: reactive follicles (red line circle), epithelioid cell clusters (histiocytes, green line circle), and monocytoid cell patches (monocyte-like cells, blue line circle); (hematoxylin-eosin stain) the third row, left: ×100, mid: ×200, and right: ×400. Case 5: reactive follicles (red line circle), epithelioid cell clusters (histiocytes, green line circle), and monocytoid cell patches (monocyte-like cells, blue line circle); (hematoxylin-eosin stain) the fourth row, left: ×20, mid: ×100, and right: ×400.

As judged by histopathological and Toxoplasma antibody assessments, the five cases were clinically diagnosed as having toxoplasmosis at our hospital (Table 1, cases 1–5; Figure 1, cases 2–5). There are no cases that showed the following subjective symptoms: fever, malaise, fatigue, and pain. The technique for measuring Toxoplasma IgG and IgM antibodies in cases 1–4 is fluorescent antibody test (FAT). The technique for measuring antibodies in case 5 is enzyme immunoassay (EIA). Avidity test was an expensive test not allowed by the Japanese health insurance systems. Patients and their families did not agree to be tested on their expenses. Suspected cases of malignant lymphoma, based on G-banding chromosome analysis, PCR (polymerase chain reaction) genetic testing, and flow cytometric cell surface marker examination, are not included in cases 2–5. Suspected cases of Epstein–Barr virus infection or cytomegalovirus infection, based on IgG and IgM antibody examinations, are not included in cases 2–5.

The details of each clinically diagnosed case are as follows:

- *Case 1*. A 28-year-old woman with no subjective symptoms but had undergone an intrauterine fetal death at 14 weeks gestation. Histopathological examination of the autopsy specimen revealed the presence of Toxoplasma cysts in the placenta, heart, adrenal gland, and brain, so this case was diagnosed as congenital toxoplasmosis [3]. Analysis of antibody levels revealed the following: phyto-hemagglutinin (PHA) antibody:20480 (<160), IgG:5120 (<20), and IgM:20 (<10). There was a serial measuring of antibodies in case 1. IgG and IgM gradually increased, peaked on the 150th day, and IgM became negative on the 310th day [5].
- *Case 2*. A 32-year-old woman had a left cervical tumor in five years. She had developed this tumor gradually increased in size recently. We resected swollen lymph nodes and examined microscopically. The lymph node findings showed reactive follicles, clusters of epithelioid cells, and patches of monocytoid cells, which is the triad of Toxoplasma lymphadenopathy [4]. IgG: 320, and IgM: 40; therefore, Toxoplasma lymphadenitis was presumed (case 2, Figure 1).
- *Case 3*. A 20-year-old woman had developed bilateral cervical tumors that had gradually increased in size over a six-month period. We resected swollen lymph nodes and examined microscopically. The lymph node findings showed reactive follicles, clusters of epithelioid cells, and patches of monocytoid cells, which is the triad of Toxoplasma lymphadenopathy [4]. IgG: 320 and IgM: 20; therefore, Toxoplasma lymphadenitis was presumed (case 3, Figure 1).
- *Case 4*. A 44-year-old woman had had right cervical tumors for three days. We resected swollen lymph nodes and examined microscopically. The lymph node findings showed reactive follicles, clusters of epithelioid cells, and patches of monocytoid cells, which is the triad of Toxoplasma lymphadenopathy [4]. IgG: 320 and IgM: 40; therefore, Toxoplasma lymphadenitis was presumed (case 4, Figure 1).
- *Case 5*. A 23-year-old man had lymphadenopathy in both posterior neck and left axilla starting one month previously. PET-CT (positron emission tomography-computed tomography) showed strong accumulation of 18F-FDG (18F-fluoro-2-deoxy-D-glucose), which was consistent with a diagnosis of malignant lymphoma. A 15 mm-sized lymph node was resected from the left axilla and examined microscopically. The lymph node findings showed reactive follicles, clusters of epithelioid cells, and patches of monocytoid cells, which is the triad of Toxoplasma lymphadenopathy [4]. Toxoplasma lymphadenitis was suspected. IgG (EIA): 45 IU/mL (less than 6), and IgM: 8.99 (cutoff index) (<0.80) (case 5, Figure 1).

## 4. Discussion

The percentage of the population infected with Toxoplasma has been decreasing along with improved hygiene in Japan [2, 3, 5, 6]. When Toxoplasma infection occurs in healthy people, it is usually mild, with lymph node enlargement being one of the most obvious clinical signs [1]. When pregnant women are newly infected with *Toxoplasma gondii*, some of the fetuses infected at this time, if they survive, can develop serious complications at birth such as blindness (chorioretinitis), mental retardation (hydrocephalus), and hepatosplenomegaly [1].

The Toxoplasma antibody positivity rate in pregnant Japanese women with a mean age of 30 was reported to be 2% in a recent investigation [6]. To reach an infection rate of 2% at 30 years old, it is necessary to infect 0.067% (2/30) of the population each year on average. With the population of Japan being 120 million and the population of Hyogo Prefecture being 5.4 million, the latter population should account for 4.5% of the population of Japan (5.4 million/120 million) [7]. Therefore, in this prefecture, we estimate that 3,600 people are infected every year: 5.4 million people × 2/30 × 1/100 = 3,600.

In our hospital, acquired Toxoplasma infections were observed in five immunocompetent patients over an 8-year period, and 0.625 (5/8) of patients were discovered annually. There are 34 maternal and fetal accreditation facilities in Hyogo Prefecture, including center facilities and supplementary facilities. Most hospitals that report Toxoplasma infections in the Japana Centra Revuo Medicina [8] (Japanese PubMed) are included in the 34 maternal and fetal accreditation facilities. These are university hospitals and perinatal center hospitals. In other words, there are 34 hospitals in Hyogo Prefecture with the same number of patients with toxoplasmosis as ours. Therefore, 21.25 people (0.625 x 34) will be discovered annually in Hyogo Prefecture..

This number represents 0.59% (21.25/3600) of all infected people. Only 10% of patients experience a symptomatic Toxoplasma infection [1]. Some patients do not attend a medical facility even when they have symptoms, and for some patients, a definitive diagnosis is not reached when their symptoms resolve spontaneously. Furthermore, some patients decline an invasive lymph node biopsy examination. Thus, the ratio of confirmed cases at 0.59% of the infected persons seems easy to understand because there are many subclinical infections that often resolve naturally. To conclude, Toxoplasma infections occurred in our hospital and in Japan rather more than expected. If we are not careful enough, there is a large possibility that we will have congenital toxoplasmosis. To prevent congenital toxoplasmosis, it is necessary for pregnant women to be educated not to eat raw meat and not to handle cat litter or cat feces or indeed soil contaminated with *Toxoplasma gondii*. It is also necessary for doctors to be educated to consider congenital toxoplasmosis as a possibility during pregnancy [9–11]. From the viewpoint of cost performance relationship, it is appropriate to pay the inspection test fee of 50 USD for the exclusion of congenital toxoplasmosis with Toxoplasma IgG and IgM at their own expenses.

## Figures and Tables

**Table 1:** One clinically diagnosed congenital toxoplasmosis case (case 1) and toxoplasmosis lymphadenopathy cases (cases 2–5) suspected to be *Toxoplasma gondii* infections based on the pathological findings and Toxoplasma antibody test.

								Toxoplasma antibody	
Case number	Sex	Age	Pathological findings					IgG (<20-fold standard value)	IgM (<10-fold standard value)

1	Female	28	A case of congenital toxoplasmosis stillbirth diagnosed at autopsy. Histopathological examination of autopsy specimen demonstrated the presence of *Toxoplasma gondii* cysts in the placenta, the heart, adrenal gland and brain.					5120	20

					Hematoxylin-eosin stain (Triad^*∗*^)				
			Lymph node location	Duration of lymphadenopathy	Reactive follicles	Epithelioid cell clusters	Monocytoid cell patches	IgG	IgM

2	Female	32	Left cervical	Increased in size recently	(+)	(+)	(+)	320	40

3	Female	20	Bilateral cervical	6 months	(+)	(+)	(+)	320	20

4	Female	44	Right cervical	3 days	(+)	(+)	(+)	320	40

5	Male	23	Bilateral postcervical, left axillar	1 month	(+)	(+)	(+)	45 IU/mL (6>)	8.99 C.O.I. (0.80>)

^*∗*^Hematoxylin-eosin stain (Triad): from the book chapter by Ioachim and Petersen. Toxoplasma Lymphadenitis p159-164. [5]. The technique for measuring antibodies in case 1–4 is fluorescent antibody test (FAT). The technique for measuring antibodies in case 5 is enzyme immunoassay (EIA). C.O.I: cut-off index. Suspected cases of malignant lymphoma, based on G-banding chromosome analysis, PCR genetic testing, and flow cytometric cell surface marker examination are not included in these four cases. Suspected cases of Epstein–Barr virus infection and cytomegalovirus infection, based on IgG and IgM antibody examination, are not included in these four cases.

## Data Availability

No data were used to support this study.
